# Protective Effects of Prophylactic and Therapeutic Erythromycin Administration in Ovarian Ischemia–Reperfusion Injury in Rats

**DOI:** 10.3390/medicina62101977

**Published:** 2026-10-13

**Authors:** Sümeyye Akyüz, Necip Gökhan Taş, Murat Günay, Nezahat Kurt, Fikret Altındağ

**Affiliations:** 1 Department of Medical Microbiology, Faculty of Medicine, Erzincan Binali Yıldırım University, Erzincan 24002, Turkey; necip.tas@erzincan.edu.tr; 2 Department of Biochemistry, Faculty of Medicine, Erzincan Binali Yıldırım University, Erzincan 24002, Turkey; mgunay@erzincan.edu.tr (M.G.); nezahat.kurt@erzincan.edu.tr (N.K.); 3 Research and Development (R&D) Laboratory, Faculty of Medicine, Erzincan Binali Yıldırım University, Erzincan 24002, Turkey; 4 Department of Histology and Embryology, Faculty of Medicine, Van Yüzüncü Yıl University, Van 65080, Turkey; fikretaltindag@yyu.edu.tr

**Keywords:** erythromycin, ovarian ischemia–reperfusion injury, oxidative stress, inflammation, ovary, rat

## Abstract

*Background and Objectives*: Ovarian ischemia–reperfusion (I/R) injury involves oxidative stress, inflammation, and structural tissue damage that may impair ovarian function. This study investigated the effects of prophylactic and therapeutic erythromycin administration on biochemical and histopathological injury in a rat model of ovarian I/R. *Materials and Methods*: Forty adult female Wistar albino rats were divided into five groups (n = 8 per group): sham (SG), I/R, erythromycin alone (E), prophylactic erythromycin plus I/R (I/R+ERP), and therapeutic erythromycin plus I/R (I/R+ERT). Ovarian ischemia was induced by clamping the right ovarian vascular pedicle for 3 h, followed by 3 h of reperfusion. Erythromycin (50 mg/kg, intraperitoneally) was administered 30 min before ischemia in the I/R+ERP group and at the onset of reperfusion in the I/R+ERT group. Inflammatory and oxidative stress markers in serum and ovarian tissue, serum anti-Müllerian hormone (AMH) levels, and ovarian hemorrhage and necrosis were evaluated. *Results:* Compared with the sham group, ovarian I/R increased inflammatory and oxidative stress markers and histopathological injury, and decreased serum AMH and ovarian tissue glutathione (GSH) and superoxide dismutase (SOD) levels. Compared with the untreated I/R group, both erythromycin regimens were associated with lower serum and ovarian tissue tumor necrosis factor-α (TNF-α) and malondialdehyde (MDA) levels and lower ovarian necrosis scores. The I/R+ERT group also had lower ovarian tissue interleukin-1β (IL-1β), higher ovarian tissue GSH and SOD levels, and a lower hemorrhage score than the untreated I/R group. In the direct comparison between the two erythromycin regimens, serum MDA was lower (false discovery rate [FDR]-adjusted *q* < 0.001), ovarian tissue SOD was higher (*q* = 0.014), and the ovarian necrosis score was lower (Holm-adjusted *p* < 0.001) in the I/R+ERT group. Direct comparisons for serum TNF-α (*q* = 0.055), ovarian tissue IL-1β (*q* = 0.127), and ovarian tissue GSH (*q* = 0.105) did not meet the FDR-adjusted significance threshold. The hemorrhage score did not differ significantly between the two erythromycin regimens (Holm-adjusted *p* = 0.057). Neither regimen significantly increased serum AMH compared with the untreated I/R group. *Conclusions*: Erythromycin attenuated several biochemical and histopathological indicators of acute experimental ovarian I/R injury. Administration at the onset of reperfusion was associated with more favorable findings than prophylactic administration for selected outcomes. These short-term findings do not establish preservation of ovarian reserve or fertility, or clinical efficacy in ovarian torsion.

## 1. Introduction

Ischemia–reperfusion (I/R) injury is a complex pathophysiological condition that develops during the restoration of blood flow following a temporary interruption of tissue perfusion [1,2]. During ischemia, inadequate oxygen and substrate availability reduce ATP production, thereby disrupting cellular energy balance, ion homeostasis, and membrane integrity [2,3]. Although restoration of blood flow is essential for preserving tissue viability, excessive production of reactive oxygen species during the early phase of reperfusion may further aggravate existing cellular injury [1,4]. Lipid peroxidation, endothelial activation, microvascular dysfunction, inflammatory cell migration, and the activation of various cell-death pathways contribute to the progression of tissue damage during this process [1,3].

Ovarian tissue is highly susceptible to interruptions in blood flow and the associated hypoxic injury. Disruption and subsequent restoration of ovarian blood flow may disturb the oxidant–antioxidant balance, enhance the inflammatory response, and impair the histological integrity of ovarian tissue. Experimental studies of ovarian I/R have reported increased levels of malondialdehyde (MDA) and proinflammatory cytokines, accompanied by reductions in antioxidant defense components such as glutathione (GSH) and superoxide dismutase (SOD) [5,6]. These biochemical alterations may be accompanied by vascular congestion, hemorrhage, interstitial edema, inflammatory cell infiltration, follicular degeneration, and apoptotic cell death [7,8]. The partial reversal of biochemical and histopathological alterations observed in studies investigating various antioxidant and anti-inflammatory agents supports the view that oxidative stress and inflammation are interrelated processes in ovarian I/R injury [9,10].

Ovarian I/R injury may affect not only acute tissue integrity but also follicular structure and ovarian reserve. Experimental studies have reported decreases in follicle counts and serum anti-Müllerian hormone (AMH) levels following ovarian I/R injury [11]. These findings provide a rationale for measuring serum AMH alongside histopathological, inflammatory, and oxidative stress outcomes in experimental ovarian I/R studies. Nevertheless, a single AMH measurement shortly after reperfusion provides limited information about the potential preservation of ovarian reserve.

In addition to their antimicrobial activity, macrolide antibiotics may exert immunomodulatory effects on cytokine production, leukocyte activation, and intracellular inflammatory signaling pathways [12]. Erythromycin, a member of this class, has been reported to reduce oxidative stress and inflammatory responses, promote neuronal cell survival, and limit ischemic tissue injury in experimental models of cerebral ischemia [13,14]. These findings suggest that erythromycin may have tissue-protective properties independent of its antibacterial activity. Nevertheless, findings obtained from other organs cannot be directly extrapolated to ovarian tissue. To the best of our knowledge, no previous experimental study has directly investigated the effects of erythromycin on ovarian I/R injury or the influence of the timing of its administration on its protective effects.

This study aimed to evaluate the effects of erythromycin on inflammation, oxidative stress, serum AMH levels, and histopathological tissue injury in a rat model of ovarian I/R induced by temporary clamping of the ovarian vascular pedicle. The effects of erythromycin administered prophylactically before ischemia or therapeutically at the onset of reperfusion were examined, and the two administration regimens were directly compared. We hypothesized that erythromycin would attenuate ovarian I/R injury when administered at either time point, but that its protective efficacy would differ by timing of administration.

## 2. Materials and Methods

### 2.1. Ethical Approval and Experimental Animals

The study was approved by the Erzincan Binali Yıldırım University Local Ethics Committee for Animal Experiments (Decision No. 28; 26 June 2025). All procedures were performed in accordance with the relevant national regulations and institutional ethical principles governing the care and use of laboratory animals.

A total of 40 adult female Wistar albino rats, aged 8–12 weeks and weighing 225–250 g, were used. The animals were housed at 22 ± 2 °C and 50–60% relative humidity under a 12 h light/12 h dark cycle, with ad libitum access to standard laboratory chow and water. A one-week acclimatization period was provided before the experiment.

Before the experiment, the rats’ estrous cycles were monitored using vaginal smears to standardize the reproductive cycle stage. Vaginal samples were examined under a light microscope, and cycle stages were determined according to their cellular characteristics. Only animals exhibiting regular 4–5-day estrous cycles were included in the study. To minimize the potential influence of cycle stage on the outcomes, all surgical procedures were performed during the diestrus phase.

### 2.2. Sample Size, Randomization, and Blinding

No a priori sample size calculation was performed. The sample size was determined based on the group sizes used in comparable experimental studies of ovarian ischemia–reperfusion and in keeping with the 3R principles (Replacement, Reduction, and Refinement), which aim to minimize animal use. Eight rats were included in each of the five groups, for a total of 40 rats. No outcome had been designated as primary or secondary before the experiment. Therefore, all outcome analyses were considered exploratory. The animals were allocated to the experimental groups using a computer-generated randomization list. Biochemical and histopathological samples were labeled with unique codes that did not disclose group allocation. Measurements and assessments were performed by investigators blinded to the group assignments.

### 2.3. Chemicals

Erythromycin lactobionate (Erythrocin^®^; Abbott GmbH & Co. KG, Wiesbaden, Germany), ketamine hydrochloride (Ketalar^®^, 50 mg/mL; Pfizer, Istanbul, Türkiye), and xylazine hydrochloride (Rompun^®^, 2%; Bayer, Istanbul, Türkiye) were used. Immediately before administration, erythromycin lactobionate was completely dissolved in sterile water for injection and subsequently diluted with sterile 0.9% NaCl solution. Erythromycin lactobionate was administered intraperitoneally at a dose of 50 mg/kg, with the injection volume calculated based on each rat’s body weight and not exceeding 0.5 mL. As a specific erythromycin dose for ovarian I/R had not been established, this dose was selected based on studies investigating a single 50 mg/kg dose of erythromycin lactobionate in rat cerebral ischemia [13,14]. However, these studies examined a different organ system and used subcutaneous administration; therefore, they cannot be used to determine the optimal dose or equivalent drug exposure in ovarian I/R. The intraperitoneal route was chosen to enable administration at the planned experimental time points. Intraperitoneal administration of erythromycin has previously been reported in a mouse experimental model [15]. The selected dose was considered an experimental dose rather than a validated ovarian dose or a clinically equivalent exposure.

### 2.4. Experimental Groups

The animals were divided into five groups, with eight rats in each group: the sham group (SG), ischemia–reperfusion group (I/R), erythromycin-alone group (E), erythromycin administered before ischemia group (I/R+ERP), and erythromycin administered at the onset of reperfusion group (I/R+ERT). The experimental groups and interventions are summarized in Table 1.

### 2.5. Anesthesia and Ovarian Ischemia–Reperfusion Model

Before surgery, general anesthesia was induced by intraperitoneal administration of ketamine hydrochloride (75 mg/kg) and xylazine hydrochloride (10 mg/kg). Approximately 3 h after the beginning of the experiment, supplemental intraperitoneal doses of ketamine (35–40 mg/kg) and xylazine (5 mg/kg) were administered to maintain anesthesia. Anesthetic depth was monitored by assessing the pedal withdrawal reflex and muscle tone.

The rats were placed in the supine position. After shaving the abdominal region, skin antisepsis was performed using povidone–iodine. Following sterile draping, a vertical midline incision approximately 2–2.5 cm in length was made in the lower abdomen. The right ovary and its vascular pedicle were carefully exposed. In the I/R groups, ischemia was induced for 3 h by applying an atraumatic microvascular clamp to the vascular pedicle containing the right ovarian artery and vein. After clamp application, discoloration and congestion of the right ovary were observed as macroscopic findings consistent with reduced ovarian perfusion. Subsequently, the ovary was returned to its anatomical position, and the abdominal incision was temporarily closed using 4–0 nylon sutures.

At the end of the 3 h ischemia period, the abdominal incision was reopened, and reperfusion was initiated by removing the clamp. After clamp removal, improvement in the color of the right ovary was observed as a macroscopic finding consistent with reperfusion. Ovarian blood flow was not measured directly. The ovary was then returned to its anatomical position, and the abdominal incision was temporarily reclosed using 4–0 nylon sutures for the duration of the reperfusion period. At the end of the 3 h reperfusion period, the abdominal cavity was reopened, and blood and right ovarian tissue samples were collected.

The same laparotomy and right ovarian exploration procedures were performed in the SG and E groups, but no clamp was applied to the vascular pedicle. The ovary was returned to its anatomical position, the abdominal incision was temporarily closed, and the animals were maintained under anesthesia for the same total experimental duration as those in the I/R groups. At the end of the 6 h period, the abdominal cavity was reopened, and blood and right ovarian tissue samples were collected.

In all groups, the excised right ovaries were divided into two portions using a sterile scalpel. One portion was fixed in 10% neutral-buffered formalin for histopathological evaluation, while the other was stored at −80 °C until biochemical analysis.

### 2.6. Erythromycin Administration

Rats in the E group received erythromycin lactobionate (50 mg/kg, i.p.) 30 min before sham surgery, and ovarian I/R was not induced in this group. Rats in the I/R+ERP group received erythromycin 30 min before the induction of ischemia, whereas those in the I/R+ERT group received erythromycin at the onset of reperfusion, immediately after removal of the vascular clamp, using the same dose and route of administration. To equalize the number of injections and injection-related stress across all groups, animals received an erythromycin-free vehicle solution in a volume equivalent to that of the active treatment at time points when erythromycin was not administered.

### 2.7. Collection and Preparation of Serum and Tissue Samples

#### 2.7.1. Serum Samples

At the end of the 3 h reperfusion period, intracardiac blood samples were collected under deep anesthesia, after which the animals were euthanized by cervical dislocation. The blood samples were allowed to clot at room temperature for 30 min and then centrifuged at 1500× *g* for 10 min. The serum was separated and stored at −80 °C until analysis. Serum samples were subsequently used to measure interleukin-1β (IL-1β), tumor necrosis factor-α (TNF-α), MDA, GSH, SOD, and AMH levels.

#### 2.7.2. Ovarian Tissue Samples

For biochemical analysis, 20 mg of ovarian tissue was weighed and combined with 1 mL of ice-cold phosphate-buffered saline (PBS; pH 7.4; LOBA Chemie Pvt. Ltd., Mumbai, India). The tissue was homogenized on ice using a homogenizer (VELP OV5 Homogenizer; VELP Scientifica, Usmate Velate, Italy) and centrifuged at 5000× *g* for 15 min at 4 °C. IL-1β, TNF-α, MDA, GSH, and SOD levels were measured in the resulting supernatant. The total protein concentration in each tissue homogenate supernatant was determined using the Bradford method [16]. The standard curve was generated using bovine serum albumin, and absorbance was measured at 595 nm. Protein concentrations were expressed in g/L and used in this unit for the calculations. Tissue biomarker levels were normalized to the total protein concentration of each sample and expressed in the corresponding measurement unit per gram of protein. All spectrophotometric measurements were performed using a Multiskan Sky Microplate Spectrophotometer (Thermo Fisher Scientific, Waltham, MA, USA).

### 2.8. Biochemical Analyses

#### 2.8.1. Study Sites and Assay Procedures

Analyses of IL-1β, TNF-α, MDA, GSH, and SOD in serum and ovarian tissue homogenates were performed at the Research and Development (R&D) Laboratory, Faculty of Medicine, Erzincan Binali Yıldırım University. Serum AMH measurements were performed at the Biochemistry Laboratory of Mengücek Gazi Training and Research Hospital. All samples were assayed in duplicate, and the mean of the two technical measurements for each animal was used for statistical analysis. Samples were coded so that the investigators performing the measurements were blinded to group allocation, as described in Section 2.2.

#### 2.8.2. Enzyme-Linked Immunosorbent Assay of IL-1β, TNF-α, MDA, GSH, and SOD in Serum and Ovarian Tissue Samples

The levels of IL-1β, TNF-α, MDA, GSH, and SOD in serum and ovarian tissue homogenates were measured spectrophotometrically using commercial ELISA kits. The kits used were as follows: Rat Interleukin-1β ELISA Kit (BT LAB; Cat. No. E0119Ra), Rat Tumor Necrosis Factor-α ELISA Kit (BT LAB; Cat. No. E0764Ra), Rat Malondialdehyde ELISA Kit (BT LAB; Cat. No. E0156Ra), Rat Glutathione ELISA Kit (BT LAB; Cat. No. EA0113Ra), and Rat Superoxide Dismutase ELISA Kit (BT LAB; Cat. No. E0168Ra).

According to the manufacturer-provided catalogs and package inserts, rat serum and tissue samples were listed as suitable sample types for all five ELISA kits. Accordingly, rat serum samples and ovarian tissue homogenates were used to measure the respective biomarkers. For each analyte, two 96-well microplates were used: one for serum samples and one for ovarian tissue homogenates. Samples from all five experimental groups were included on each corresponding plate, so the groups were not analyzed on separate plates. Kit standards were included on each plate, and concentrations were calculated from the standard curve generated on that plate.

The measurements of IL-1β, TNF-α, MDA, and SOD were based on the sandwich ELISA principle. Standards, serum samples, and ovarian tissue homogenates were added to microplate wells pre-coated with antibodies specific to the respective analytes. Following binding of the analytes to the immobilized antibodies, analyte-specific biotinylated detection antibodies and streptavidin–horseradish peroxidase (streptavidin–HRP) were used. After the addition of the substrate, the color reaction was allowed to develop and was then terminated with stop solution. Absorbance was measured at 450 nm.

GSH was measured using a competitive ELISA. In this method, GSH present in the samples competes with biotinylated GSH antigen for binding. Following color development, the reaction was terminated by adding stop solution, and absorbance was measured at 450 nm. GSH concentrations were calculated using the standard curve generated from the kit standards. The resulting signal was inversely proportional to the GSH concentration.

The analytical sensitivity values reported by the manufacturers were 0.08 ng/mL for IL-1β, 2.51 ng/L for TNF-α, 0.01 nmol/mL for MDA, 10.25 mg/L for GSH, and 0.022 ng/mL for SOD. According to the manufacturer’s specifications, the intra-assay and inter-assay coefficients of variation (CVs) were <8% and <10%, respectively.

The ELISA-derived tissue biomarker concentrations were divided by the corresponding protein concentrations (g/L) for normalization. Before this calculation, the volume units were converted to a common denominator of liters (L) to eliminate differences between the denominators of the ELISA result units and the protein concentration units. Following protein normalization, where necessary, the resulting numerical values were converted to equivalent higher or lower units to ensure clear and appropriate reporting; these conversions did not alter the biomarker amount, only the unit of expression. The final tissue results were expressed as ng/g protein for TNF-α, IL-1β, and SOD; as mg/g protein for GSH; and as nmol/g protein for MDA.

#### 2.8.3. Serum AMH Analysis

Serum AMH concentrations were measured using a rat AMH ELISA kit (Hangzhou Eastbiopharm Co., Ltd., Hangzhou, China; Cat. No. CK-E90200) according to the manufacturer’s instructions. Serum samples from all five experimental groups were distributed across two AMH assay microplates, with each group represented on both plates. Kit standards were included on each plate, and concentrations were calculated using the standard curve generated on that plate. Absorbance was measured at 450 nm using a microplate reader, and AMH concentrations were expressed as ng/mL. The analytical sensitivity and measurement range were 0.05 ng/mL and 0.1–40 ng/mL, respectively.

### 2.9. Histopathological Evaluation

Ovarian tissues were fixed in 10% neutral-buffered formalin and embedded in paraffin following routine tissue processing. Sections measuring 5 µm in thickness were obtained from the paraffin blocks at 30 µm intervals between the starts of successive sampled sections and stained with hematoxylin and eosin (H&E). The sections were examined under a light microscope (Olympus BX53; Tokyo, Japan).

The ovarian sections were examined for morphological changes. Hemorrhage and necrosis were selected for semiquantitative scoring because they were the most prominent changes observed across the experimental groups; both have been assessed in previous rat ovarian I/R studies [17]. For each of five ovarian sections per animal, ten non-overlapping microscopic fields at ×200 magnification were identified and numbered. One field per section was selected using a random number generator, yielding five evaluated fields per animal. Hemorrhage and necrosis were scored separately using a semiquantitative scale ranging from 0 to 3, based on the proportion of tissue affected within each evaluated microscopic field: 0 (−), absent; 1 (+), mild (<25%); 2 (++), moderate (25–50%); and 3 (+++), severe (>50%). A comparable four-grade approach has been used to score hemorrhage and necrosis separately in a rat I/R model [18]. The five field scores were averaged separately for hemorrhage and necrosis to obtain one score for each outcome per animal. Thus, each animal was treated as a single independent observation in the statistical analysis. Coded slides were examined in random order by a single experienced histopathologist who was blinded to group allocation. Because only one histopathologist performed the scoring, interobserver agreement was not assessed.

### 2.10. Statistical Analysis

The normality of continuous variables was assessed using the Shapiro–Wilk test, and homogeneity of variances was evaluated using Levene’s test. Biochemical outcomes were analyzed using one-way analysis of variance (ANOVA), followed by Tukey’s honestly significant difference (HSD) post hoc test. For serum SOD, the homogeneity-of-variance assumption was not met; therefore, Welch’s ANOVA and the Games–Howell post hoc test were used. The overall between-group *p*-value was reported for each biochemical outcome. The four pairwise comparisons—SG versus I/R, I/R versus I/R+ERP, I/R versus I/R+ERT, and I/R+ERP versus I/R+ERT—had been selected before the experiment in accordance with the study objectives. This advance selection concerned the group comparisons, rather than the designation of primary or secondary outcomes. These comparisons were evaluated for each biochemical marker, regardless of whether its overall test was statistically significant. Their post hoc *p*-values were further adjusted using the Benjamini–Hochberg false discovery rate (FDR) procedure across all 11 serum and ovarian tissue biomarkers (44 comparisons in total). The resulting *q*-values are reported in Table 2 and Table 3, with *q* < 0.05 used to interpret pairwise biochemical findings. For selected biochemical outcomes, mean differences between the I/R+ERP and I/R+ERT groups and their unadjusted 95% confidence intervals (CIs) were reported to describe the magnitude and precision of the direct between-regimen comparison; statistical significance was interpreted using the corresponding *q*-values. For serum GSH and SOD, 95% CIs for the comparisons of each erythromycin regimen with the I/R group were obtained using Tukey HSD and Games–Howell, respectively. For serum AMH, Tukey HSD-adjusted 95% CIs were also reported for the comparisons of each erythromycin regimen with the I/R group.

Overall between-group comparisons of hemorrhage and necrosis scores were performed using the Kruskal–Wallis test. The same four pairwise group comparisons described above were conducted using two-sided exact permutation Mann–Whitney U tests. The family-wise type I error arising from the four pairwise comparisons was controlled using the Holm method. For the direct comparison of hemorrhage scores between the two erythromycin regimens, the probability that an I/R+ERT animal had a lower score than an I/R+ERP animal was also calculated, with ties assigned half weight. A descriptive, unadjusted 95% CI for this effect measure was obtained by resampling animals within each group using the percentile bootstrap method. Statistical significance was assessed using the Holm-adjusted *p*-value.

Parametric data are presented as the mean ± standard deviation. Although histopathological scores are descriptively presented as the mean ± standard deviation, nonparametric tests were used for statistical comparisons because of the ordinal nature of the scores. All tests were two-sided. Holm-adjusted *p*-values were used for histopathological pairwise comparisons. Biochemical analyses were performed using IBM SPSS Statistics version 25.0 (IBM Corp., Armonk, NY, USA) and Python version 3.12; FDR adjustments and confidence intervals were calculated using Python. Exact permutation tests and Holm corrections for the histopathological scores were also performed using Python.

## 3. Results

No deaths or exclusions occurred during the experiment. All 40 animals completed the study protocol and were included in the analyses.

### 3.1. Biochemical Findings

#### 3.1.1. Serum Biochemical Findings

Table 2a presents serum biomarker levels across the five experimental groups. Serum TNF-α, IL-1β, and MDA levels were higher in the I/R group than in the SG group (all FDR-adjusted *q* < 0.001). Serum SOD was lower in the I/R group than in the SG group (*q* = 0.032), whereas serum GSH did not differ significantly between these groups (*q* = 1.000). Serum AMH was lower in the I/R group than in the SG group (*q* < 0.001). Serum TNF-α and MDA levels were lower in both the I/R+ERP and I/R+ERT groups than in the I/R group (all *q* < 0.001). Serum IL-1β did not differ significantly between the I/R group and either erythromycin regimen (Table 2b).

For serum GSH, the mean differences relative to the I/R group were 2.03 mg/L for I/R+ERP (Tukey HSD 95% CI, −95.78 to 99.84; FDR-adjusted *q* = 1.000) and 2.76 mg/L for I/R+ERT (Tukey HSD 95% CI, −95.04 to 100.57; *q* = 1.000). For serum SOD, the corresponding mean differences were 0.178 ng/mL for I/R+ERP (Games–Howell 95% CI, −0.114 to 0.470; *q* = 0.451) and 0.229 ng/mL for I/R+ERT (Games–Howell 95% CI, −0.224 to 0.682; *q* = 0.592). Serum AMH also did not differ significantly from the I/R group in either the I/R+ERP group (mean difference [I/R+ERP − I/R], 0.0115 ng/mL; Tukey HSD 95% CI, −0.0081 to 0.0311; FDR-adjusted *q* = 0.592) or the I/R+ERT group (mean difference [I/R+ERT − I/R], 0.0191 ng/mL; Tukey HSD 95% CI, −0.0005 to 0.0388; *q* = 0.105; Table 2b).

In the direct comparison between erythromycin regimens, serum MDA was lower in the I/R+ERT group than in the I/R+ERP group (mean difference [I/R+ERT − I/R+ERP], −0.288 nmol/mL; unadjusted 95% CI, −0.314 to −0.261; FDR-adjusted *q* < 0.001). Serum TNF-α was also numerically lower (mean difference, −3.16 ng/L; unadjusted 95% CI, −5.50 to −0.82), but this comparison did not meet the FDR-adjusted significance threshold (*q* = 0.055). Serum AMH did not differ significantly between the regimens (mean difference, 0.008 ng/mL; unadjusted 95% CI, −0.002 to 0.017; FDR-adjusted *q* = 0.950). Serum IL-1β, GSH, and SOD also did not differ significantly between the regimens (Table 2b).

#### 3.1.2. Ovarian Tissue Biochemical Findings

Ovarian tissue TNF-α, IL-1β, and MDA levels were higher, whereas GSH and SOD levels were lower, in the I/R group than in the SG group (all FDR-adjusted *q* < 0.001). Ovarian tissue biomarker levels in the E group were numerically similar to those in the SG group. Compared with the I/R group, the I/R+ERP group had lower TNF-α (*q* < 0.001) and MDA (*q* = 0.003) levels. The corresponding comparisons for IL-1β, GSH, and SOD did not meet the FDR-adjusted significance threshold (*q* = 0.232, *q* = 0.337, and *q* = 1.000, respectively). Compared with the I/R group, the I/R+ERT group had lower TNF-α, IL-1β, and MDA levels and higher GSH and SOD levels (all *q* < 0.05; Table 3a).

In the direct comparison between erythromycin regimens, ovarian SOD was higher in the I/R+ERT group than in the I/R+ERP group (mean difference [I/R+ERT − I/R+ERP], 138.75 ng/g protein; unadjusted 95% CI, 58.65 to 218.85; FDR-adjusted *q* = 0.014). Ovarian IL-1β was numerically lower (mean difference, −264.58 ng/g protein; unadjusted 95% CI, −447.44 to −81.72; *q* = 0.127), and GSH was numerically higher (mean difference, 5.49 mg/g protein; unadjusted 95% CI, 3.47 to 7.50; *q* = 0.105), but neither comparison met the FDR-adjusted significance threshold. Ovarian TNF-α and MDA also did not differ significantly between the regimens (*q* = 0.950 and *q* = 0.337, respectively; Table 3a,b).

### 3.2. Histopathological Findings

**Figure 1:**
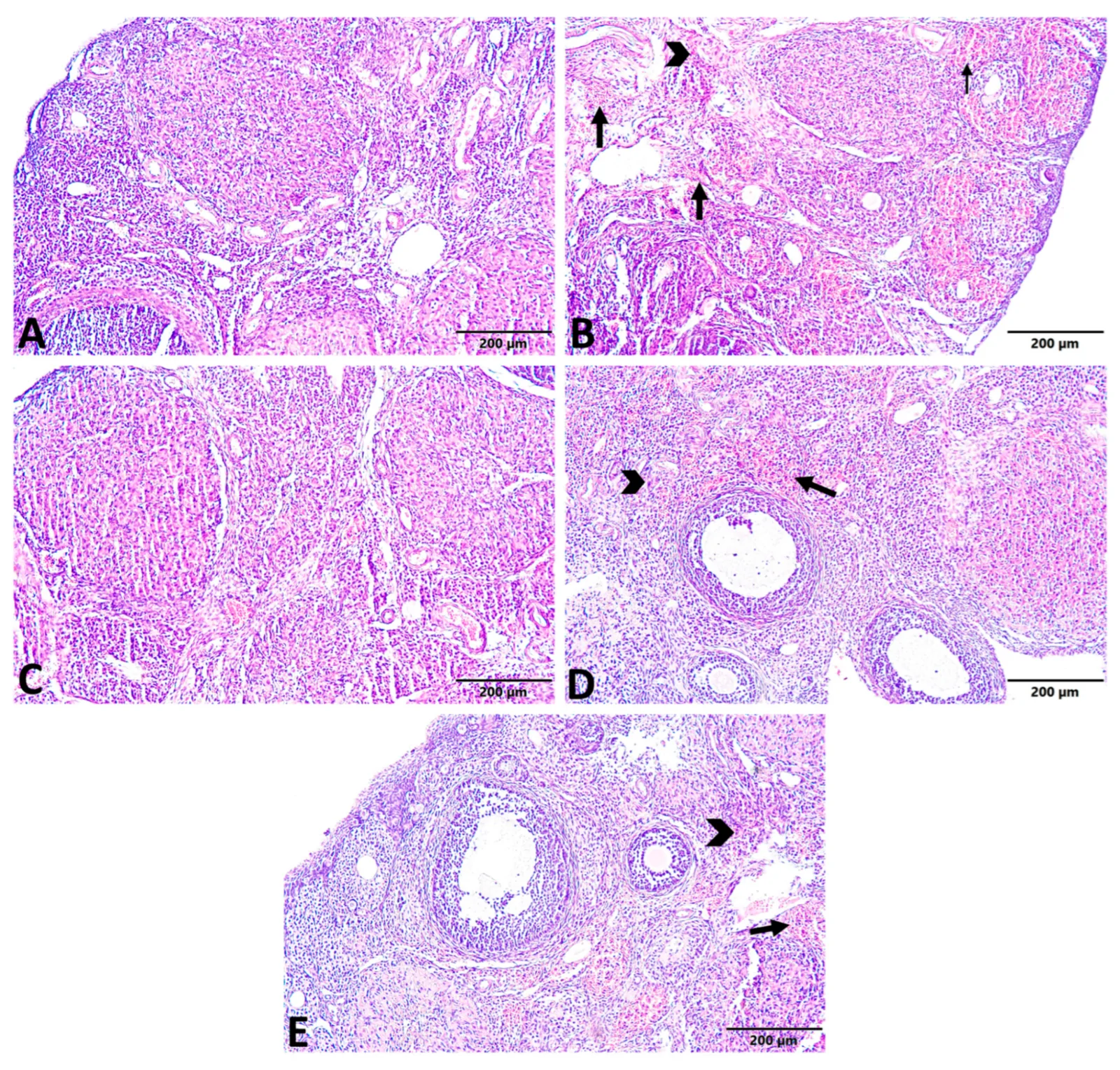
Representative H&E-stained ovarian sections from the (**A**) sham, (**B**) ischemia–reperfusion, (**C**) erythromycin-alone, (**D**) prophylactic erythromycin plus I/R, and (**E**) therapeutic erythromycin plus I/R groups. Areas of intense hemorrhage (arrow) and necrotic cells (arrowhead) were observed in the ischemia–reperfusion group. These pathological changes were reduced in groups (**D**,**E**). Original magnification: ×200; scale bars: 200 µm.

In the SG and erythromycin-alone (E) groups, ovarian tissue architecture was generally preserved, with minimal hemorrhagic and necrotic changes. In contrast, extensive hemorrhage and marked necrotic cellular injury were observed in the I/R group (Figure 1).

Hemorrhage and necrosis scores were significantly higher in the I/R group than in the SG group (*p* < 0.001 for both). The hemorrhage score did not differ significantly between the I/R+ERP and I/R groups (*p* = 0.076), whereas the necrosis score was lower in the I/R+ERP group than in the I/R group (*p* < 0.001).

**Figure 2:**
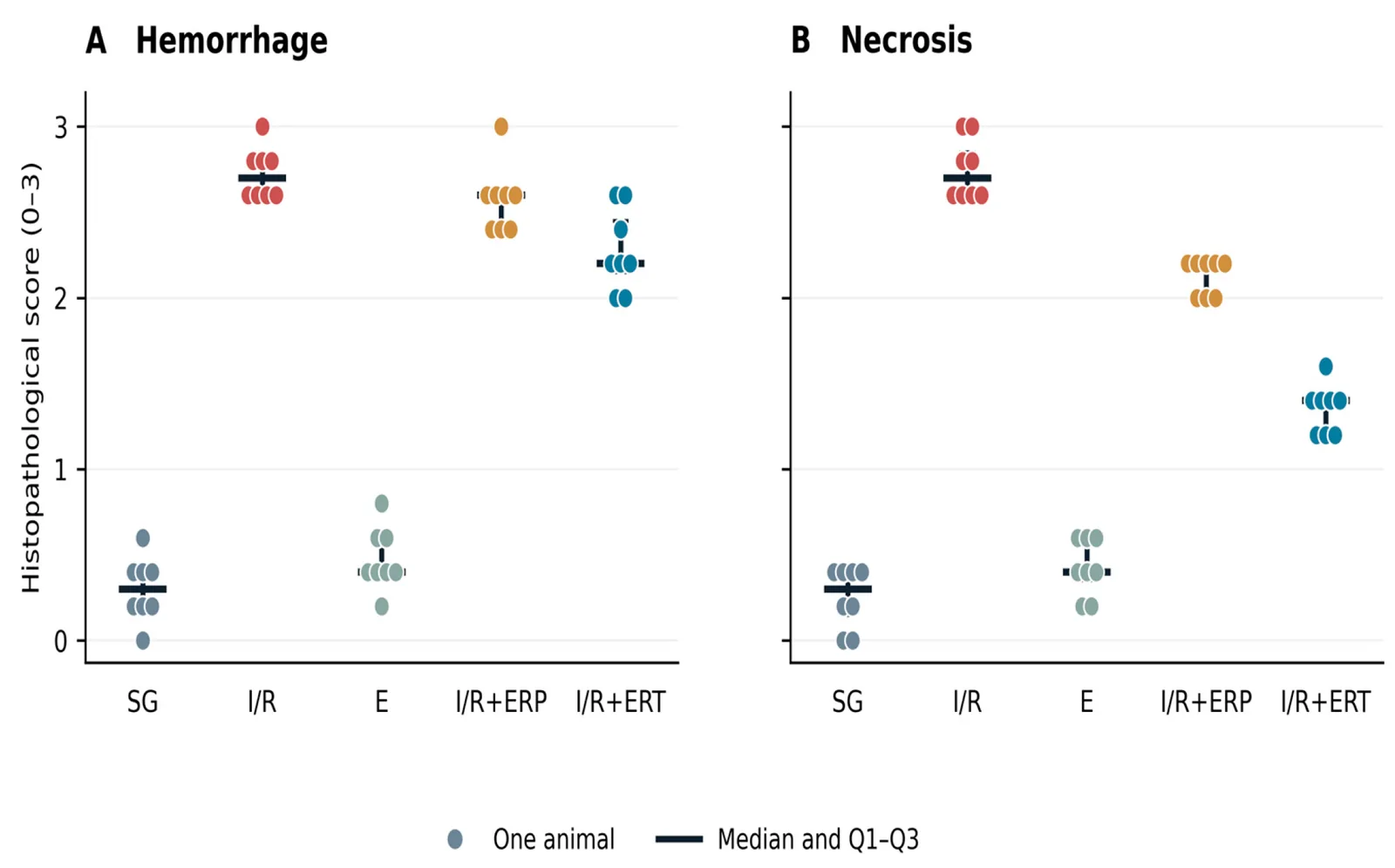
Distribution of ovarian histopathological scores among the experimental groups. (**A**) Hemorrhage scores and (**B**) necrosis scores. Each point represents the mean score of five microscopic fields from one animal (n = 8 per group). Horizontal black lines indicate the median, and vertical black bars indicate the first and third quartiles (Q1–Q3). Hemorrhage and necrosis scores were highest in the I/R group. Both erythromycin regimens had lower necrosis scores than the I/R group; the hemorrhage score was significantly lower than that of the I/R group only in the I/R+ERT group. SG, sham group; I/R, ischemia–reperfusion group; E, erythromycin-alone group; I/R+ERP, prophylactic erythromycin plus I/R group; I/R+ERT, therapeutic erythromycin plus I/R group.

In the I/R+ERT group, in which erythromycin was administered at the onset of reperfusion, hemorrhage and necrosis scores were significantly lower than those in the I/R group (*p* = 0.007 and *p* < 0.001, respectively). When the two erythromycin regimens were compared directly, the necrosis score was significantly lower in the I/R+ERT group than in the I/R+ERP group (*p* < 0.001). The hemorrhage score did not differ significantly between the I/R+ERP and I/R+ERT groups (*p* = 0.057; Table 4 and Figure 2). In this direct comparison, the estimated probability that an I/R+ERT animal had a lower hemorrhage score than an I/R+ERP animal was 0.820 (descriptive, unadjusted bootstrap 95% CI, 0.594–0.992); the Holm-adjusted comparison remained nonsignificant (*p* = 0.057).

## 4. Discussion

The present study demonstrated that ovarian ischemia–reperfusion increased the inflammatory response and lipid peroxidation, reduced antioxidant defense, and caused marked histopathological injury. Both prophylactic and therapeutic administration of erythromycin attenuated some of these alterations. In the direct comparison, the I/R+ERT group had lower serum MDA, higher ovarian tissue SOD, and a lower necrosis score than the I/R+ERP group; however, these selected differences do not establish overall superiority across the evaluated outcomes. These results suggest that erythromycin may reduce certain indicators of acute ovarian I/R injury. Nevertheless, the differences between the regimens cannot be attributed solely to the timing of administration.

Oxidative stress is a major component of ovarian I/R injury. Ingec et al. demonstrated increased MDA levels and DNA damage, together with decreased GSH and SOD levels, in a vascular clamp-induced model of ovarian I/R [19]. Studies involving curcumin and melatonin have similarly reported increased lipid peroxidation and oxidative burden following I/R [20,21]. Furthermore, pomegranate seed oil and trimetazidine have been shown to reduce MDA levels and help preserve antioxidant capacity [22,23]. In the present study, the increase in ovarian tissue MDA levels and the decrease in GSH and SOD levels in the I/R group were consistent with previous experimental findings. The increased serum and ovarian tissue levels of TNF-α and IL-1β in the I/R group indicate that an inflammatory response accompanied ovarian injury. JZL184 has been reported to attenuate inflammation, oxidative stress, and apoptosis by reducing TNF-α and IL-1β levels and nuclear factor kappa B (NF-κB) activity [24]. A study involving benidipine similarly demonstrated that reductions in proinflammatory cytokines were accompanied by improvements in oxidative, histopathological, and immunohistochemical findings [25]. Collectively, these findings suggest that oxidative stress and inflammation are interrelated processes that reinforce each other during ovarian I/R injury.

Serum and ovarian tissue TNF-α levels were lower in both erythromycin-treated groups than in the I/R group. Previous studies in other experimental settings have reported effects of erythromycin on NF-κB- and activator protein-1 (AP-1)-related inflammatory signaling, adhesion molecule expression, and leukocyte migration [26,27,28]. Uchimura et al. also reported attenuation of proinflammatory responses following erythromycin administration [29]. These findings provide possible biological context for the present observations; however, none of these pathways or cellular processes were measured in the present study. Therefore, the mechanism responsible for the observed TNF-α differences cannot be established.

Ovarian tissue IL-1β levels were lower in the I/R+ERT group than in the I/R group (FDR-adjusted *q* < 0.001), whereas serum IL-1β levels did not differ significantly between these groups (*q* = 0.757). Neither serum nor ovarian tissue IL-1β differed significantly between the I/R+ERP and I/R groups (*q* = 0.592 and *q* = 0.232, respectively). In the direct comparison of the two erythromycin regimens, ovarian tissue IL-1β was numerically lower in the I/R+ERT group, but the difference did not meet the FDR-adjusted significance threshold (*q* = 0.127). These differing serum and tissue findings may be due to variations in the timing or intensity of local and systemic inflammatory responses. In a mouse model of intestinal I/R, rapamycin further increased IL-1β in intestinal tissue without a corresponding additional increase in serum IL-1β [30]. Although that study examined a different organ and intervention, it illustrates why a tissue IL-1β finding need not be accompanied by a corresponding serum finding. In the present study, serum and ovarian tissue IL-1β levels were measured at a single time point; therefore, the temporal course of these responses and the mechanisms underlying their differing patterns could not be determined.

The oxidative stress findings differed according to the timing of administration. Prophylactic erythromycin reduced serum and ovarian tissue MDA levels but did not significantly increase GSH or SOD levels. Therapeutic administration reduced serum and ovarian tissue MDA levels while increasing ovarian tissue GSH and SOD levels. In the direct comparison of the two regimens, the I/R+ERT group had lower serum MDA (FDR-adjusted *q* < 0.001) and higher ovarian tissue SOD (*q* = 0.014) than the I/R+ERP group. Ovarian tissue GSH was numerically higher in the I/R+ERT group, but this difference did not meet the FDR-adjusted significance threshold (*q* = 0.105). These differences were observed in selected markers of the oxidant–antioxidant balance; the present design does not establish whether they resulted from administration timing or differences in drug exposure before sampling. Likewise, the present findings do not establish that erythromycin acts as a direct free-radical scavenger or increases antioxidant enzyme synthesis. Erythromycin may have indirectly limited secondary reactive oxygen species production by reducing the inflammatory response. Similarly, experimental studies involving astaxanthin, glycyrrhetinic acid, and chrysin have shown that reduced oxidative stress is accompanied by preservation of antioxidant capacity and histopathological improvement [31,32]. Findings obtained with rosmarinic acid, vitamin D, and oxytocin also support the association between limiting oxidative injury and preserving ovarian tissue integrity [33,34,35]. The serum GSH and SOD comparisons with the I/R group were not statistically significant, but their 95% confidence intervals included both decreases and increases. Thus, these findings do not establish the absence of an effect on circulating antioxidant markers or permit a robust comparison of the magnitudes of serum and ovarian tissue responses. Furthermore, because serial measurements, antioxidant enzyme activities, and molecular signaling pathways were not evaluated, a definitive mechanism cannot be proposed.

Hypoxia-responsive signaling and apoptosis may also contribute to ovarian I/R injury. Previous experimental studies of ovarian I/R have examined hypoxia-inducible factor-1α (HIF-1α) and reported changes in apoptosis-related proteins, including Bcl-2, Bax, and caspase-3 [36,37]. As these pathways were not assessed in the present study, the observed effects of erythromycin cannot be attributed to them.

The histopathological results supported the biochemical findings. Extensive hemorrhage and marked necrosis were observed in the I/R group, whereas necrosis scores were reduced in both erythromycin-treated groups. Therapeutic administration also reduced the hemorrhage score compared with the I/R group. Previous studies have demonstrated that ovarian I/R can result in hemorrhage, congestion, edema, follicular degeneration, and necrosis [19,20,25]. Studies involving astaxanthin and rosmarinic acid have also reported that biochemical improvement was accompanied by reduced histopathological injury [31,33]. In the direct comparison, the necrosis score was lower in the I/R+ERT group than in the I/R+ERP group (*p* < 0.001). The hemorrhage score did not differ significantly between these groups (*p* = 0.057). Given the small group size and the variation in individual hemorrhage scores, this nonsignificant comparison should not be interpreted as evidence that the two regimens have equivalent effects on hemorrhage. Results from controlled reperfusion and JZL184 studies also support the potential importance of the early reperfusion period and treatment timing in determining the protective response [19,24].

As ovarian torsion is generally unpredictable, administration before ischemia would rarely be feasible in clinical practice. Early diagnosis and timely surgical detorsion remain central to the management of ovarian torsion [38]. In the present study, erythromycin was administered either before vascular pedicle clamping or at the onset of reperfusion. The regimens differed in both administration timing and the interval between drug administration and tissue sampling. Therefore, the observed differences cannot be attributed solely to treatment timing. Furthermore, vascular pedicle clamping does not fully replicate clinical ovarian torsion, and the outcomes were evaluated after only 3 h of reperfusion. These findings relate to specific acute biochemical and histopathological outcomes in this experimental model and do not demonstrate the therapeutic efficacy of erythromycin in patients with ovarian torsion.

Serum AMH was lower in the I/R group than in the SG group (FDR-adjusted *q* < 0.001), which may reflect an acute response to ovarian injury. Experimental studies have reported decreases in AMH levels and follicle counts following ovarian I/R [24,39,40]. Serum AMH did not differ significantly between the I/R group and either the I/R+ERP or I/R+ERT group (*q* = 0.592 and *q* = 0.105, respectively), or between the two erythromycin regimens (*q* = 0.950). The 95% confidence intervals for both comparisons with the I/R group included zero; given the small group size, these nonsignificant findings do not exclude a possible effect on serum AMH. In a clinical study of 11 patients undergoing laparoscopic ovarian detorsion, Yasa et al. measured serum AMH and antral follicle counts before surgery and at one and three months postoperatively. They found no significant change in serum AMH during follow-up, and no significant difference in antral follicle counts between the operated and contralateral ovaries at three months [41]. In the present study, AMH was measured only once, after 3 h of reperfusion; follicle counts, serial AMH measurements, and long-term fertility outcomes were not assessed. Therefore, the present findings cannot establish whether either erythromycin regimen preserves ovarian reserve or fertility.

The strengths of this study include the evaluation of prophylactic and therapeutic erythromycin administration within the same experimental model, the direct comparison of the two administration regimens, the inclusion of an erythromycin-alone control group, and the simultaneous assessment of serum and ovarian tissue biomarkers. Standardization of the estrous cycle, blinded biochemical and histopathological evaluations, and analysis of each animal as an independent experimental unit further strengthened the study’s methodological rigor. Nevertheless, this study has several limitations. An a priori sample size calculation was not performed, and only a single erythromycin dose, a single ischemia–reperfusion duration, and a single terminal sampling time were evaluated. The 50 mg/kg dose was extrapolated from studies of cerebral ischemia, and neither a dose–response analysis nor measurements of erythromycin concentrations in serum or ovarian tissue were conducted. Therefore, the optimal dose for ovarian I/R and the pharmacological relevance of the exposure in this model remain unknown. The small group size (n = 8) limits the precision of the estimates. Although the four pairwise group comparisons were selected before the experiment, no outcome was designated as primary or secondary. The outcome analyses therefore remain exploratory. Testing multiple biochemical outcomes and pairwise comparisons increases the risk of type I error. The Benjamini–Hochberg false discovery rate procedure was applied to the reported biochemical pairwise comparisons to address this multiplicity; nevertheless, the findings should be confirmed in future studies. Consequently, nonsignificant findings, particularly those relating to serum AMH, should not be interpreted as evidence of no effect.

Molecular signaling pathways, antioxidant enzyme activities, apoptosis markers, follicle counts, long-term changes in AMH levels, and fertility outcomes were not evaluated. Although rat tissue samples were listed as suitable by the kit manufacturers, additional analytical validation specifically for ovarian tissue homogenates, such as dilutional linearity or spike-and-recovery testing, was not performed. Although ovarian sections were examined for morphological changes, only hemorrhage and necrosis were scored semiquantitatively, limiting quantitative assessment of other features of ovarian injury. The 0–3 histopathological scoring scale was not independently validated for this study. Because only one microscopic field was scored from each of five ovarian sections per animal, focal changes elsewhere in those sections may not have been captured.

Ischemia and reperfusion were assessed based on macroscopic changes in ovarian color rather than on direct measurements of ovarian blood flow. Consequently, the extent of perfusion reduction and restoration, and its consistency across animals, could not be objectively verified. As the interval between erythromycin administration and tissue sampling differed between the two treatment groups, differences between the regimens cannot be attributed solely to the timing of administration. Furthermore, the short reperfusion period limits assessment of longer-term outcomes, and vascular pedicle clamping does not fully replicate clinical ovarian torsion. These limitations restrict the applicability of the findings to patients with ovarian torsion.

## 5. Conclusions

Both erythromycin regimens were associated with improvements in selected biochemical and histopathological indicators of acute experimental ovarian I/R injury compared with the untreated I/R group. In the direct comparison between regimens, administration at the onset of reperfusion was associated with lower serum MDA, higher ovarian tissue SOD, and a lower ovarian necrosis score than prophylactic administration. The other reported direct biochemical comparisons and the hemorrhage score did not differ significantly after the respective multiple-comparison adjustments. These findings do not establish the overall superiority of either regimen.

Neither erythromycin regimen significantly increased serum AMH compared with the I/R group. The short observation period and the absence of long-term reproductive outcomes mean that these findings do not establish preservation of ovarian reserve or fertility, or therapeutic efficacy in patients with ovarian torsion. Further studies are needed to compare doses and administration times, to investigate potential mechanisms, and to assess long-term ovarian function and fertility.

## Figures and Tables

**Table 1:** Experimental Groups and Procedures (n = 8/group).

Group	Description	Procedure
SG	Sham	Sham laparotomy; no I/R or erythromycin administration
I/R	Ischemia–reperfusion	3 h of ischemia followed by 3 h of reperfusion
E	Erythromycin alone	Erythromycin (50 mg/kg, i.p.) alone; no I/R procedure
I/R+ERP	Prophylactic erythromycin + I/R	Erythromycin (50 mg/kg, i.p.) administered 30 min before ischemia; 3 h of ischemia followed by 3 h of reperfusion
I/R+ERT	Therapeutic erythromycin + I/R	Erythromycin (50 mg/kg, i.p.) administered at the onset of reperfusion; 3 h of ischemia followed by 3 h of reperfusion

i.p., intraperitoneal.

**Table 2:** (**a**) Serum biomarker levels; (**b**) Overall and Pairwise Statistical Comparisons of Serum Biomarkers.

**Panel (a)**					
	**Experimental Groups (n = 8/Group)**				
**Biomarkers**	**SG (1)**	**I/R (2)**	**E (3)**	**I/R+ERP (4)**	**I/R+ERT (5)**
TNF-α (ng/L)	30.57 ± 2.39	50.56 ± 1.59	31.54 ± 1.80	39.13 ± 2.46	35.97 ± 1.82
IL-1β (ng/mL)	2.20 ± 0.24	4.20 ± 0.32	2.85 ± 0.43	3.94 ± 0.24	3.98 ± 0.32
MDA (nmol/mL)	1.23 ± 0.05	1.82 ± 0.05	1.31 ± 0.02	1.68 ± 0.03	1.39 ± 0.02
GSH (mg/L)	465.03 ± 61.92	438.80 ± 58.88	450.07 ± 34.93	440.83 ± 86.48	441.57 ± 25.23
SOD (ng/mL)	1.41 ± 0.10	1.15 ± 0.12	1.23 ± 0.21	1.32 ± 0.17	1.37 ± 0.28
AMH (ng/mL)	0.339 ± 0.026	0.300 ± 0.010	0.325 ± 0.006	0.311 ± 0.007	0.319 ± 0.010
**Panel (b)**					
**Biomarkers**	**Overall** ***p***	**1 vs. 2** ***q***	**2 vs. 4** ***q***	**2 vs. 5** ***q***	**4 vs. 5** ***q***
TNF-α (ng/L)	**<0.001**	**<0.001**	**<0.001**	**<0.001**	0.055
IL-1β (ng/mL)	**<0.001**	**<0.001**	0.592	0.757	1.000
MDA (nmol/mL)	**<0.001**	**<0.001**	**<0.001**	**<0.001**	**<0.001**
GSH (mg/L)	0.930	1.000	1.000	1.000	1.000
SOD (ng/mL)	0.032 *	**0.032**	0.451	0.592	1.000
AMH (ng/mL)	**<0.001**	**<0.001**	0.592	0.105	0.950

(**a**): Data are presented as mean ± standard deviation. SG, sham group; I/R, ischemia–reperfusion group; E, erythromycin-alone group; I/R+ERP, prophylactic erythromycin plus I/R group; I/R+ERT, therapeutic erythromycin plus I/R group. (**b**): Overall *p*-values were obtained using one-way ANOVA, except for serum SOD (*), for which Welch’s ANOVA was used because the homogeneity-of-variance assumption was not met. Pairwise *q*-values were obtained by applying the Benjamini–Hochberg false discovery rate procedure to Tukey HSD post hoc *p*-values (Games–Howell for serum SOD) across the four reported comparisons for all 11 serum and ovarian tissue biomarkers in Table 2 and Table 3 (44 comparisons). Bold values indicate statistical significance (overall *p* < 0.05; pairwise *q* < 0.05).

**Table 3:** (**a**): Ovarian Tissue Biomarker Levels. (**b**): Overall and Pairwise Statistical Comparisons of Ovarian Tissue Biomarkers.

**Panel (a)**					
	**Experimental Groups (n = 8/Group)**				
**Biomarkers**	**SG (1)**	**I/R (2)**	**E (3)**	**I/R+ERP (4)**	**I/R+ERT (5)**
TNF-α (ng/g protein)	102.63 ± 10.83	153.89 ± 14.06	108.35 ± 7.00	128.47 ± 7.66	122.81 ± 8.00
IL-1β (ng/g protein)	1681.46 ± 169.95	3010.02 ± 247.46	1520.57 ± 103.78	2776.98 ± 135.34	2512.40 ± 148.34
MDA (nmol/g protein)	938.44 ± 66.79	1310.09 ± 46.65	930.65 ± 66.49	1158.80 ± 100.68	1083.13 ± 72.06
GSH (mg/g protein)	44.87 ± 6.36	28.91 ± 1.52	41.71 ± 2.93	33.08 ± 1.40	38.57 ± 1.70
SOD (ng/g protein)	1492.50 ± 72.25	1177.14 ± 51.69	1395.30 ± 70.50	1212.70 ± 42.23	1351.45 ± 73.77
**Panel (b)**					
**Biomarkers**	**Overall** ***p***	**1 vs. 2** ***q***	**2 vs. 4** ***q***	**2 vs. 5** ***q***	**4 vs. 5** ***q***
TNF-α (ng/g protein)	**<0.001**	**<0.001**	**<0.001**	**<0.001**	0.950
IL-1β (ng/g protein)	**<0.001**	**<0.001**	0.232	**<0.001**	0.127
MDA (nmol/g protein)	**<0.001**	**<0.001**	**0.003**	**<0.001**	0.337
GSH (mg/g protein)	**<0.001**	**<0.001**	0.337	**<0.001**	0.105
SOD (ng/g protein)	**<0.001**	**<0.001**	1.000	**0.001**	**0.014**

(**a**): Data are presented as mean ± standard deviation. SG (1), sham group; I/R (2), ischemia–reperfusion group; E (3), erythromycin-alone group; I/R+ERP (4), prophylactic erythromycin plus I/R group; I/R+ERT (5), therapeutic erythromycin plus I/R group. (**b**): Overall *p*-values were obtained using one-way ANOVA. Pairwise *q*-values were obtained by applying the Benjamini–Hochberg false discovery rate procedure to Tukey HSD post hoc *p*-values for the four reported comparisons across the 11 serum and ovarian tissue biomarkers in Table 2 and Table 3 (44 comparisons). Bold values indicate statistical significance (overall *p* < 0.05 or pairwise *q* < 0.05).

**Table 4:** Histopathological Scores and Pairwise Comparisons among the Experimental Groups.

	Experimental Groups (n = 8/Group)					Pairwise Comparison *p*-Value			
Histopathological Status	SG (1)	I/R (2)	E (3)	I/R+ERP (4)	I/R+ERT (5)	1 vs. 2	2 vs. 4	2 vs. 5	4 vs. 5
Hemorrhage	0.30 ± 0.19	2.73 ± 0.15	0.48 ± 0.18	2.58 ± 0.20	2.28 ± 0.24	**<0.001**	0.076	**0.007**	0.057
Necrosis	0.25 ± 0.18	2.75 ± 0.18	0.43 ± 0.17	2.13 ± 0.10	1.35 ± 0.14	**<0.001**	**<0.001**	**<0.001**	**<0.001**

Data are presented descriptively as mean ± standard deviation. Histopathological findings were scored semiquantitatively on a scale of 0–3: 0 (−), absent; 1 (+), mild; 2 (++), moderate; and 3 (+++), severe. The mean score from five microscopic fields was calculated for each animal. Overall group comparisons were performed using the Kruskal–Wallis test, and pairwise comparisons were performed using exact permutation-based Mann–Whitney U tests with Holm adjustment. Adjusted *p*-values < 0.05 were considered statistically significant and are shown in bold. SG, sham group; I/R, ischemia–reperfusion group; E, erythromycin-alone group; I/R+ERP, prophylactic erythromycin plus I/R group; I/R+ERT, therapeutic erythromycin plus I/R group.

## Data Availability

The original contributions presented in this study are included in the article. Further inquiries can be directed to the corresponding author.
